# Osteopathy Redivivus

**Published:** 1906-03

**Authors:** 


A Monthly Review of Medicine and Surgery.

*EDITOR:*

WILLIAM WARREN POTTER, M. D.

All communications, whether of a literary or business nature, books for review and exchanges, should be addressed to the editor 284 FRANKLIN ST., BUFFALO, N. Y.

VOL. LXI. MARCH, 1906. No. 8

### Osteopathy Redivivus.

WITH the return of the legislative season the Osteopath, with a zeal worthy of a loftier purpose, pays his annual pilgrimage to Albany, to endeavor to persuade the law makers to clothe him with the legal title of "Doctor." Were it not that a serious side to the question exists, these attempts to hoodwink the legislature into conferring a dignified professional status upon a group of uneducated manipulators, would partake of the nature of opera bouffe.

It is quite surprising to the average observer that the honorable members of the New York legislature will permit themselves to listen, year after year, to the fustian of these masseurs and masseuses, who plead to be permitted to evade the very law that the legislature has so carefully and wisely put upon the statute books. It would be well if the members of both houses would read the address of Vice-Chancellor McKelway, published elsewhere in this issue, noting in particular what he says under the subheads, "The State Made Doctor," and "The Function of the Board of Regents." We quite agree with the learned Vice-Chancellor that there should be no step backward in reference to medical education in this State.

The worst feature of the present bill is embodied in the following excerpt: Sec. 6.

"Any person, who at the time of the passage of this act shall be actually engaged in the practice of Osteopathy in this state, and who is a graduate in good standing of a regularly conducted School of Osteopathy within the United States requiring a course of two years or longer, with actual attendance of at least twenty months, *and who shall be recommended to the Regents by the state board of Osteopathic Examiners,* shall upon application and payment of twenty-five dollars, without examination, be granted a license to practise Osteopathy, provided application for such license be made within six months after the passage of this act."

It is an absurdity to admit to the practise of medicine, for that is what is really intended, several hundred persons without examination, none of whom has exhibited satisfactory evidence of preliminary education in accordance with the established standard of the education departments. The italics in the quoted section of the pending bill are our own, made for the purpose of inviting attention to this particularly objectionable feature of it. If the osteopaths would ask the legislature to license them after meeting the tests now applied under the law, no possible objection could exist to such a bill. It would be a manly way to obtain recognition which would deserve and receive all praise. When, therefore, the osteopath asserts that the profession of medicine is opposed to him or desires to oppose him, he asserts that which is untrue. What every educated physician objects to is the method by which this class of persons seeks to acquire the title of "doctor," and not the individuals themselves, nor to their mode of practice.

It is proper, however, for us to point out the absurdity of the claims of osteopathy to be able to readjust dislocated vertebrae, and thus restore to health the afflicted. If the legislative committees to whom the osteopaths appeal, and others who are asked to accept this impossibility as a truth, will reflect a moment on the difficulty of dislocating the vertebrae of the neck during the process of hanging, the absurdity of the claim will become apparent. If it is so difficult in the neck under the conditions named, how utterly impossible to dislocate the dorsal or lumbar vertebrae except through the gravest traumatism which causes, in general, death or paralysis below the point of injury. Again, if it be next to impossible to dislocate the vertebrae, how still more difficult to readjust the bones; no touch, no rub, no simple manipulation can possibly accomplish it, and only in rare exceptions can the highest exercise of surgical skill succeed. No person in the world with a knowledge of anatomy could be deceived by any such claim as is made in behalf of osteopathy in regard to vertebral displacement.

In discussing the several phases of this subject there is one which must not be overlooked, because in certain quarters an attempt has been made to exaggerate its bearing to a successful antagonism of the bill. Our esteemed contemporary, "*The Post Graduate*," has presented this point in a recent issue so well that we are constrained to print its remarks.

Very much has been said of late about the importance of the unity of the profession in the State of New York, in order to confront the Osteopaths and Opticians who desire to enter the profession of Medicine without a proper training. It has been thought by some that the existence of three Boards, one for the regular Profession of Medicine, one for Homeopathists and one for the Eclectics, was a warrant for granting similar Boards to the Osteopaths and Opticians. We cannot conceive that this is in the slightest respect true. If the Osteopaths and the Faith Curers and the Opticians will do what is demanded from the Homeopaths, the Regulars and the Eclectics, that is, spend four years in the study of medicine and pass an examination in all departments of anatomy, physiology, medicine, chemistry and surgery, the State will be very willing, through the Regents of the University, to grant them special examinations in therapeutics.

The *Post Graduate* is quite right. There is no relation between these state examining boards and opposition to osteopathy. It is quite possible that some astute or cunning members of the legislature have set up the excuse that successful resistance to the demands of the osteopaths cannot be made until the examining boards become united in a single body. This subterfuge seems to have been taken seriously by some persons, particularly in the western end of the state. Even a so-called "legislative committee" has been instructed to "prepare a bill" looking to the consolidation of the boards; whereas, in this state, under the wise administration of the Education Department, though nominally there are three boards, yet practically there is but one; hence this talk of the bearing of the three board system upon the demands of the osteopaths is idle. There may be good reason for the consolidation of the boards, but this is not one of them. When the three several "Schools" of Medicine became consolidated into one compact body, — united in Medical Colleges and Medical Societies, — then the state examining boards will, perforce, be merged into one body. The union of the three divisions of medicine in this state cannot be forced by an appeal to law; it must result from an enlightened public sentiment in which the homeopathic and eclectic branches must take the lead. Union by legislative enactment will not prove satisfactory to any of the contracting parties.

The Medical Society of the State of New York made a *manifest mistake* in 1883-4 when it sought to create a State Medical examining board without consulting the constituent societies; this error caused some years delay in the enactment of the medical practice law. Whenever the other State Medical Societies desire union they will intimate the wish to the Medical Society of the State of New York and the latter will be only too glad to meet them half way. Meanwhile let us continue,—jointly if possible, separately if need be,—to oppose any amendment to the present medical law that is offered by unincorporated medical bodies or individuals.

The osteopaths, according to the press dispatches, have been holding "high jinks" recently in Trenton, which is in the State of New Jersey. At a legislative hearing held March 12, 1906, there was a large attendance in favor of and opposed to the measure. There were some twenty-five women who were said to be living illustrations of the effects of osteopathic treatment.

This is a new feature at these "hearings" which hereafter will deserve to be called "seeings," if this method shall become a fixed plan. Possibly the next move will be to give a clinical exhibition of the "treatment" to the legislatures, which would certainly draw a large crowd.

At Trenton, C. W. Proctor, of Buffalo, according to the news reports, was the leading speaker for the bill. He explained what osteopathy was—training or manipulation of the parts of the body to keep the circulation, the liver and the nerves in normal condition. He asserted that manipulation in most cases was better than medicine. He then told of the growth of the school. In nearly every state, he said, the practice was recognised by the courts and the legislature. He said the school was well established in twenty-seven states. He asked that New Jersey allow the practice to be legally established. We cannot learn, however, that Mr. Proctor informed the Committee on Public Health that the Buffalo institution, called the "Atlantic School of Osteopathy," in which he had been a "professor," was put out of commission by the State Education Department for illegalities in its charter and in the conduct of its affairs. All that seemed to be lacking to make the opera bouffe at Trenton complete, was the presence of that "lightning change artist" Martin W. Littleton, whose attempt at Albany a few days previously to lend variety to the "doings" was certainly a success.

---

MR. EDWARD H. BUTLER, editor and proprietor of the *Buffalo Evening News,* has subscribed $5,000.00 to the University of Buffalo extension fund. This generous and timely subscription gives encouragement to the work, that is advancing steadily under the leadership of Vice-Chancellor Norton, who is supported by an active staff of coadjutors. It is an object that every citizen should have near his heart and encourage by word, deed, and money according to his ability. Let the good work go forward with reasonable speed.

---

CALOX, the oxygen dentifrice,—the ideal tooth powder,—is steadily growing in favor with physicians and dentists. As they become more familiar with its chemistry and with the clinical results that follow its daily use, they unhesitatingly recommend it to their patients. It will soon supplant every other form of tooth powder with persons of education and refinement, who know the destructive power of pathogenic mouth germs, and who desire a safe and efficient, as well as agreeable, destroyer of these dangerous microorganisms.

---

The Types played many pranks with us in the last previous issue of this magazine, most of which were the fault of the composer and proof reader at the printing house. One related to the name of one of the orators at the centenary of the Medical Society of the State of New York. We wrote Biggs and the composer made it read "Briggs." We think, however, he will not again assume to know better than the editor how to spell the proper names we print in these pages.

